# Editorial: Traditional medicine bioactives for management of diabetes mellitus

**DOI:** 10.3389/fphar.2023.1205939

**Published:** 2023-06-06

**Authors:** Pukar Khanal, Rupesh Chikhale, Yadu Nandan Dey, Mutiu Idowu Kazeem

**Affiliations:** ^1^ Department of Pharmacology and Toxicology, KLE College of Pharmacy Belagavi, KLE Academy of Higher Education and Research (KAHER) Belagavi, Belagaum, India; ^2^ Department of Pharmaceutical and Biological Chemistry, UCL School of Pharmacy, University College London, London, United Kingdom; ^3^ Department of Pharmacology, Dr. B.C. Roy College of Pharmacy and Allied Health Sciences, Durgapur, India; ^4^ Department of Biochemistry, Faculty of Science, Lagos State University, Ojo, Nigeria

**Keywords:** diabetes mellitus, ethnobotanicals, hyperglycemia, polygenic disease, traditional medicines

Diabetes mellitus is a chronic metabolic disorder characterized by a hyperglycemic state due to the body’s inability to produce (type 1) or properly use (type 2) insulin. Other than type 1 and 2, some specific diabetes due to other etiological factors include monogenic diabetes syndromes (neonatal diabetes), diseases of the exocrine pancreas (pancreatitis), and chemical (glucocorticoid)/virus (enteroviruses)-induced diabetes. In addition, gestational diabetes is not readily overt before gestation but can be discovered in the second or third trimester of pregnancy. Natural products, such as extracts of ethnobotanicals, have been used for centuries to treat multiple ailments, including diabetes. Various attempts are being made to isolate bioactive metabolites from the ethnobotanicals and to investigate their effect on hyperglycemic state and insulin sensitivity in patients with diabetes.

One of the commonly used ethnobotanicals, *Lonicerae Japonicae Flos* extract and its active biomolecule (chlorogenic acid), was evaluated as a potential anti-diabetic agent in a prediabetic rat model. Therein authors reported the ameliorating effect of extract and chlorogenic acid on lipid and glucose homeostasis, followed by the upregulation of adiponectin receptors and enhanced activity of downstream AMPK in the liver. Furthermore, the efficacy of the extract in increasing serum C1q tumor necrosis factor-related protein (CTRP) 3 and 9 levels, as well as chlorogenic acid potency in increasing serum CTRP3 and peroxisome proliferator-activated receptor-α (PPAR-α) expression, were reported. This study indicates that *L. Japonicae* and chlorogenic acid modulate blood sugar and lipid levels and prevent prediabetes *via* the CTRPs- AdipoRs-AMPK/PPAR axis (Guo et al.).

The ameliorative effect of bayberry leaves proanthocyanidins (BLPs) in the *Drosophila melanogaster* model by examining phenotypical, biochemical, and molecular parameters related to diabetes was also reported. In addition, authors reported the *a*-amylase and *a*-glucosidase inhibitory effects of BLPs that are directly concerned with post-prandial hyperglycemia. Further, they suggest the downstream regulation of dAKT-dFOXO-PEPCK*,* together with E78, SREBP, FAS, and LSD genes after BLPs treatment which is directly concerned with insulin signaling (Wang et al.).

The beneficial effect of mulberry leaf in managing diabetes was also reported. It possesses an important role in diabetes management which is evidenced through experimental and clinical studies. This might be due to the composition of multiple secondary bioactive metabolites like alkaloids, flavonoids, polyphenols, polysaccharides, sterols, volatile oils, and vitamins that directly contribute to diabetes management followed by the inhibition of the *a*-amylase and *a*-glucosidase to ameliorate postprandial hyperglycemia. Its protective effect on the pancreatic *ß*-cells, amelioration of oxidative stress, minimizing insulin resistance, and acceleration of peripheral glucose utilization are also summarized (Chen et al.).

One of the clinical trials investigated the multiple effects of cinnamon (500 mg of cinnamon, 3 × days), ginger (500 mg of ginger, 3 × days), and metformin (500 mg of metformin 3 × days) intake to ameliorate sex hormones, lipid profiles, insulin level and fasting blood glucose and anthropometric indices in 100 women (17 were excluded, 83 were considered for data analysis) with polycystic ovarian syndrome for 8 weeks. It was suggested that cinnamon supplementation, like metformin, reduced insulin resistance and testosterone levels. Furthermore, ginger supplementation ameliorated luteinizing and follicle-stimulating hormone, which was not observed with metformin treatment (Dastgheib et al.).

One of the active principles from *Gymnema sylvestre*, gymnemagenin*,* was investigated for its potential in promoting lipid metabolism using a series of computational and wet-lab experiments. Herein, authors reported the efficacy of gymnemagenin in ameliorating triglyceride metabolism by enhancing the lipase genes, i.e., Lipe and Lpl*.* In addition, gymnemagenin was observed to downregulate Plin2, Cidea, and Scd1, which are associated with adipogenesis, and upregulate PPAR-γ. Moreover, the binding affinity of gymnemagenin towards PPAR-γ was also reported, which is one of the targets for diabetes treatment (DasNandy et al.).

The studies in this Research Topic demonstrate the promise of a few natural compounds (chlorogenic acid and gymnemagenin) as well as natural extracts, with a strategy to treat diabetes mellitus.

